# Angelman syndrome presenting with a rare seizure type in a patient with 15q11.2 deletion: a case report

**DOI:** 10.1186/s13256-015-0622-8

**Published:** 2015-06-16

**Authors:** Jagath C. Ranasinghe, Damitha Chandradasa, Sanjaya Fernando, Uditha Kodithuwakku, D.E.N. Mandawala, Vajira HW Dissanayake

**Affiliations:** 1 Anuradhapura Teaching Hospital, Anuradhapura, Sri Lanka; 2 Asiri Center for Genomic and Regenerative Medicine, University of Colombo, Colombo, Sri Lanka; 3 Human Genetics Unit, Faculty of Medicine, University of Colombo, Colombo, Sri Lanka

**Keywords:** Angelman syndrome, 15q11.2 deletion, Flexor and extensor spasms

## Abstract

**Introduction:**

Angelman syndrome, a neurodevelopmental genetic disorder associated with abnormalities in chromosome15q11-q13, is inherited from the mother. Epilepsy is seen in 85 % of children with Angelman syndrome within the first 3 years of life and is often severe and difficult to control.

**Case presentation:**

We report a case of a baby boy who presented at 13 months of age with a history of acute gastroenteritis and marked gross motor and speech developmental delay. He was found to have a microdeletion of the chromosome 15q11.2 region confirming the diagnosis of Angelman syndrome. He was the first child born to healthy, unrelated Sinhalese parents. The child had generalized extensor spasms involving both upper limbs and the head beginning at the age of 9 months, and he developed flexor and extensor spasms at the age of 13 months. His facial appearance was characteristic of Angelman syndrome. His electroencephalographic pattern did not correspond to any other of the patterns previously described in patients with Angelman syndrome. He had extensor and flexor spasms, which are rarely described in patients with Angelman syndrome. These symptoms responded to a combination of valproic acid and clonazepam.

**Conclusions:**

Angelman syndrome due to a microdeletion of the chromosome 15q11.2 region is often not diagnosed in infancy. Extensor and flexor spasms are not typically described seizure types in Angelman syndrome, and our patient’s seizures responded well to a combination of valproic acid and clonazepam. Clinicians should suspect other possible seizure types in patients with Angelman syndrome and should treat the patient appropriately.

## Introduction

Angelman syndrome (AS), a neurodevelopmental genetic disorder associated with abnormalities in chromosome 15q11-q13, is inherited from the mother. These abnormalities are a de novo interstitial deletion of 15q11-q13 (70 % to 75 % of patients), maternal uniparental disomy of chromosome 15 (2 % of patients), imprinting center mutations (2 % of patients) and mutations in ubiquitin protein ligase E3A gene, *UBE3A* (5 % of patients). In 10 % of patients with AS, the genetic etiology is unidentified [1].

The condition is now increasingly being diagnosed worldwide because of improvement of diagnostic tests [2]. The estimated incidence of AS ranges from 1 in 10,000 to 1 in 40,000 in different populations [3].

Epilepsy is seen in 85 % of children with AS within the first 3 years of life and is often severe and difficult to control [4]. Different types of seizures and four characteristic electroencephalographic (EEG) patterns have been described in patients with AS [5].

## Case presentation

A 13-month-old baby boy was presented to our hospital with a history of acute gastroenteritis and marked gross motor and speech developmental delay. He was the first child born to healthy, unrelated Sinhalese parents. The mother was 23 years old, and the father was 21 years old. The mother noted the child having abnormal movements involving the head and the upper limbs at 9 months of age, the description of which resembled generalized extensor spasms. There was clustering of similar events and sleep deprivation, which were compatible with seizures, from that age onward.

The initial generalized extensor spasms involving both upper limbs and the head, occurring as short-lasting clusters (one spasm would persist for about 1 to 2 seconds, with recurrence in 10 to 20 clustering events, each occurrence lasting for about 3 to 5 minutes), transformed by the age of 1 year and 2 months into spasms of a flexor type resembling infantile spasms. Subsequently, the child developed generalized tonic-clonic convulsions. Most of the time, the seizures were precipitated by a low-grade fever, leading to repeated hospital admissions. The focus for the recurrent fever was not identified.

The seizures could not be controlled with valproic acid (VPA) (30mg/kg body weight/day) alone for 1 month and in combination with carbamazepine (CBZ) (5mg/kg body weight/day) for 2 weeks. VPA administered together with clonazepam (CNZ) (0.5mg/kg body weight/day), after omission of CBZ, proved to be effective, and the child remained seizure-free for 4 months. There were no early side effects of the drugs noted at a follow-up examination.

**Fig. 1:**
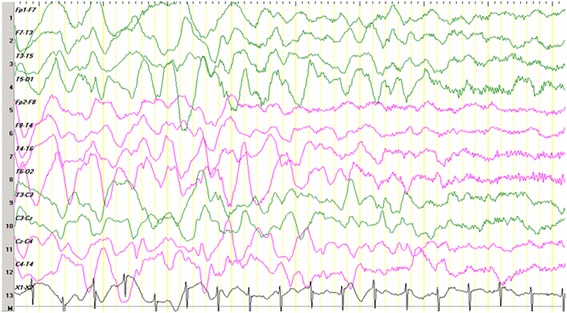
Electroencephalogram obtained when the patient was 13 months of age. We noted an immature sleep background composed of blocks of diffuse, moderate-amplitude, polymorphic delta activities alternating with a delta-theta mixed background. No epileptiform discharges were observed

**Fig. 2:**
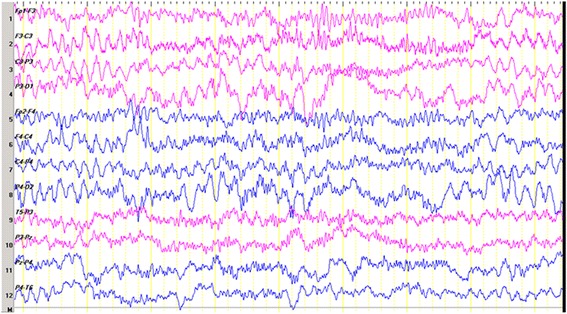
Normal sleep electroencephalogram after 2 months of treatment, with age-matched background with symmetric sleep spindles and a few K complexes. Excess diffuse fast activity could likely be a clonazepam-induced phenomenon

A physical examination revealed that the child had marked hypotonia with characteristic facial appearance of deep-set eyes, a wide mouth with protruding tongue and a pointed nose resembling AS. He is fair in complexion, with a hypopigmented patch over the left anterior thigh. Characteristically, he keeps his hands upheld, and he exhibits abnormal, uncoordinated, jerky movements of the arms. He always tends be seated with a rounded back, suggestive of underlying scoliosis, and he has episodes of inappropriate laughter. His teeth had not yet erupted, and he had marked developmental delay affecting all components. When he presented to our hospital at the age of 13 months, all developmental parameters resembled those of a child between 7 and 8 months of age. Monosyllabic babbling had started at the age of 1 year. After 4 months of treatments, he showed marked improvement. There were no seizures. He was able to sit without support and able to keep his back straight. He was stable in standing position with support for a very short period (<5s). He had weight gain. The abnormalities observed on the first EEG tracing (Fig. 1) were not characteristic of AS. The sleep background was immature and comprised of blocks of diffuse, moderate-amplitude, polymorphic delta activities alternating with a delta-theta mixed background. No epileptiform discharges were seen. Repeat EEG scan (Fig. 2) done after 2 months of treatment revealed a normal sleep EEG scan with age-matched background with symmetric sleep spindles, as well as a few K complexes. Excess diffuse fast activity could likely be a CNZ-induced phenomenon.

Computed tomography of the brain showed prominent sulci with widened subarachnoid spaces bilaterally in the frontal region, and a few hypodense areas were seen in the frontal white matter, predominantly in the periventricular region and centrum semiovale. Furthermore, there was mild prominence in the ventricular system favoring early cerebral atrophy.

His karyotype at 500-band resolution was that of a normal male (46,XY). Microdeletion testing by multiplex ligation probe amplification (MLPA) using the SALSA^®^ MLPA^®^ P290-B2 Prenatal Deletions probemix (MRC-Holland, Amsterdam, the Netherlands) according to the manufacturer’s protocol showed the presence of a deletion involving all the probes for the 15q11.2 region.

## Discussion

AS was first described in three unrelated children as “happy puppet” syndrome by English pediatrician Harry Angelman in 1965 [6]. Later, in 1982, Williams and Frias documented the natural history of AS [7].

Characteristic features of AS are frequent paroxysms of laughter, abnormal puppet-like gait, ataxia, characteristic facies, intellectual disability, seizures, sleep disturbances and a characteristic behavioral profile with delay in developmental milestones with learning disabilities and language impairment [2, 3, 6, 7]. The majority of patients with AS experience some form of seizures during their lifetime. Confirmation of the diagnosis during infancy is not common [8].

Flexion and tonic extension-type seizures are rarely described in AS. The most frequent types of seizures are atypical absence, generalized tonic-clonic, atonic and myoclonic seizures. In a series of 35 patients described by Galván-Manso and colleagues, only 6 (17 %) had extensor spasms and 2 (6 %) had flexion-type seizures [9]. Of patients with 15q11-q13 deletion, 50 % experienced atypical absence, generalized tonic-clonic, atonic or myoclonic seizures [8]. Although only 25 % develop seizures in the first year of life [10], the majority (85 %) develop seizures by the age of 3 years [4]. Early diagnosis and treatment of seizures in our patient were achieved, with successful control of seizures and improved developmental milestones over a period of 4 months. This observation about AS is new, because the majority (85 %) of cases present in later life and the seizures are difficult to control [4]. Early genetic confirmation helps in successful management.

Patients with a chromosome 15q11.2 deletion experience intellectual disability, speech and language delay, motor delay, autistic spectrum disorder, epilepsy and schizophrenia [11]. There are a significant number of patients with idiopathic generalized epilepsy who have subsequently been confirmed to have a chromosome 15q11.2 deletion [12].

In 1988, three EEG patterns were described in a patient with AS by Boyd and colleagues [13]. In 2003, four EEG patterns were described by Valente and co-workers (a hypsarrhythmia-like variant; an ill-defined, slow, spike-and-wave variant; a triphasic–like variant; and a slow variant) [5]. Our patient, however, did not show any of these EEG patterns.

VPA and CNZ can effectively control seizures in patients with AS when these agents are used as monotherapy. Some patients, however, require a combination of VPA and CNZ or VPA and clobazam [8, 11].

Our patient’s seizures were controlled poorly with CBZ. A subsequent change to a combination of VPA and CNZ resulted in good seizure control.

## Conclusions

AS due to a microdeletion of chromosome 15q11.2 region is often not diagnosed in infancy. Extensor and flexor spasms are not typically described seizure types in AS, and our patient’s seizures responded well to a combination of VPA and CNZ. Even though there were characteristic EEG patterns, his EEG scans did not correspond to any of the EEG patterns described previously in AS. However, in a patient with AS, other EEG patterns also should be considered. Clinicians should suspect other possible seizure types in patients with AS and should treat them appropriately.

## Consent

Written informed consent was obtained from the child’s parent for publication of this case report and any accompanying images. A copy of the written consent is available for review by the Editor-in-Chief of this journal.

## Abbreviations

AS: Angelman syndrome
CBZ: carbamazepine
CNZ: clonazepam
EEG: electroencephalographic
MLPA: multiplex ligation probe amplification
VPA: valproic acid
